# Temporary Gutter Endoleak to Reduce Renovisceral Ischemia During Urgent In Situ Laser Fenestrated Paravisceral Aortic Aneurysm Repair: Technical Note

**DOI:** 10.1177/15266028251333565

**Published:** 2025-04-22

**Authors:** Vaiva Dabravolskaite, Annarita Santoro, Giuseppe Asciutto, Anders Wanhainen, Marek Kuzniar, Kevin Mani

**Affiliations:** 1 Section of Vascular Surgery, Department of Surgical Sciences, Uppsala University, Uppsala, Sweden; 2 Department of Vascular Surgery, University Hospital of Bern, Bern, Switzerland; 3 Department of Vascular Surgery, San Raffaele Scientific Institute, Vita-Salute University School of Medicine, Milan, Italy; 4 Department of Surgical and Perioperative Sciences, Surgery, Umeå University, Umeå, Sweden

**Keywords:** in situ laser fenestration, superior mesenteric artery, visceral ischemia, gutter endoeak, urgent procedure, endovascular aneurysm repair, complex abdominal aortic aneurysm

## Abstract

**Purpose:**

Emergent repair of paravisceral aortic aneurysms (pAAs) with in situ laser fenestration (ISLF) technique is associated with renovisceral ischemia. We describe two strategies of temporary gutter endoleak creation to ensure renovisceral perfusion during ISLF.

**Technique:**

Two patients (79- and 83-year-old) presented with symptomatic pAA. A Cook Zenith Alpha endograft (Cook Medical LLC, Bloomington, IN, USA) was used in both cases. All visceral vessels were prestented to serve as guide markers. Prior to the deployment of the endograft, a Cook Flexor 8F-sheath (Cook Medical LLC, Bloomington, IN, USA) was placed in the superior mesenteric artery (SMA) (patient #1), and a 5 mm × 200 mm angioplasty Armada balloon (Abbott, Green Oaks, Illinois, USA) was placed between the endograft and the aortic wall (patient #2). Angiograms after visceral coverage confirmed perfusion of the renovisceral arteries through the intentional gutter endoleaks. Thereafter, ISLF and bridging stenting for SMA and the renal arteries were performed before the removal of the sheath or balloon to stop the gutter endoleak. Both patients did not experience any kind of perioperative complications.

**Conclusions:**

The above-described techniques for gutter endoleak creation during emergent pAA repair with ISLF can potentially reduce reno visceral ischemia and increase the ISLF technique’s safety.

**Clinical Impact:**

The ISLF technique for pAA repair typically requires coverage of the visceral arteries with unpredictable ischemia time until laser fenestrations are established. The gutter endoleak technique reduces the renovisceral ischemia and potentially increases the safety of the ISLF repair.

## Introduction

In situ laser fenestration (ISLF)^1^ in emergency cases involving the paravisceral or thoracoabdominal aorta has shown to be technically feasible and with reported postoperative mortality and reintervention rates comparable to standard fenestrated—endovascular aortic repair (F-EVAR).^2^Fare clic o toccare qui per immettere il testo.

However, ISLF repair of paravisceral aortic aneurysms (pAAs) bears with itself a temporary covering of the renovisceral arteries prior to the creation of laser fenestrations, resulting in potentially disastrous complications.^3–5^ Several refinements of the original technique^6^ to address this issue have been described. The use of the fusion technique and the prestenting of the visceral target vessels help in reducing the cannulation time, thus minimizing visceral ischemia.^6^ Ad hoc single fenestration custom-made devices (CMDs), based on the Cook Zenith platform (Cook Medical LLC, Bloomington, IN, USA), ensure continuous perfusion to at least one target vessel during ISLF.^3^

We describe two techniques based on the principle of creating a gutter endoleak between tubular endografts and the aortic wall during ISLF for pAA, thus minimizing visceral ischemic time.

## Technique

### Case Presentation

#### Patient #1

A 79-year-old male who previously underwent an infrarenal EVAR presented with a symptomatic expanding 10 cm pAA due to a type IA endoleak. The patient was considered unfit for open repair. Off-the-shelf branched endografts were regarded as not appropriate due to the need for extensive aortic coverage, with increased risk for spinal cord ischemia. An urgent 4-vessel ISLF procedure was planned with a proximal landing in a healthy descending aorta, and distal landing in the main body of the previously implanted infrarenal stentgraft.

#### Patient #2

An 83-year-old male who previously underwent an infrarenal EVAR with a 40-mm CMD (Cook Medical LLC, Bloomington, IN, USA) presented with a symptomatic 11 cm pAA with a type IA endoleak. Due to radiologic and clinical signs of imminent rupture, an urgent endovascular treatment was planned with a 4-vessel ISLF landing in the main body of the previously implanted infrarenal stent graft.

### Technique

#### Patient #1

The procedure was performed in a hybrid angiosuite (Artis Pheno; Siemens AG, Forchheim, Germany) under fusion guidance and general anesthesia with bilateral percutaneous femoral access.

All visceral vessels were cannulated and prestented with Visi-Pro Balloon-Expandable Stents (Medtronic, Minneapolis, MN, USA) to serve as a guiding marker. An 8F-Flexor sheath (Cook Medical LLC, Bloomington, IN, USA) was introduced and advanced in the SMA over a Rosen guidewire (Cook Medical LLC, Bloomington, IN, USA) and left in position to provide continuous perfusion to SMA through a retrograde gutter endoleak along the sheath during the ISLF procedure. The position of the sheath was toward the left aspect of the aorta across the left renal artery ostium area. A sheath was used in this case to allow for shunting of flow to the SMA via connection to the contralateral groin if needed (eg, if the gutter endoleak was not deemed adequate). Under fusion imaging, the endograft (Cook Medical LLC, Bloomington, IN, USA) was advanced through the contralateral groin and deployed over the ostium of the visceral vessels. The proximal extension stentgraft was sized to reach aortic wall and, hence, reduce the risk for gap-related endoleak. The 18F delivery system was exchanged for a 16F Dryseal sheath (Gore W.L. Gore and Associates). A 7.5F Aptus steerable sheath (7.5F, 55-mm Aptus; Medtronic) was placed inside the Dryseal and a 1.7-mm Turbo Elite laser catheter (Philips Healthcare) was advanced through the sheath.

**Figure 1.**
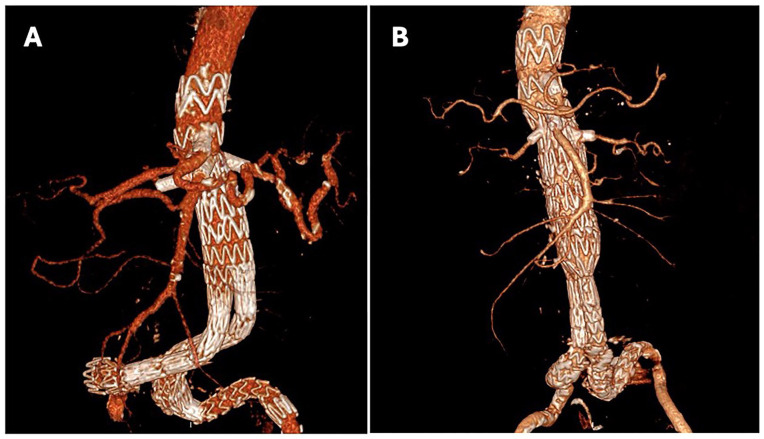
Postoperative follow-up imaging of (A) patient 1 and (B) patient 2 in 3D reconstruction, showing the proximal extension of the repair to supraceliac aorta with in situ laser fenestration technique.

An angiogram via the Dryseal sheath showed complete perfusion of at least SMA and left renal artery (LRA) retrograde through the gutter endoleak along the 8F sheath (Supplemental Video S1). The ISLF was performed using the standard technique as described before.^6^ The first vessel to revascularize was the right renal artery (RRA), followed by the celiac trunc (CT), SMA, and LRA. The 8F sheath was removed before the SMA ISLF was performed. Completion angiography and cone beam computed tomography (CBCT) showed well-expanded and patent visceral bridging stents without signs of endoleak of any kind. The postoperative course was uneventful, without any change of the creatinine levels nor any raise in lactate postoperatively. The 1-month control computed tomography showed complete aneurysm exclusion (Figure 1A).

#### Patient #2

**Figure 2.**
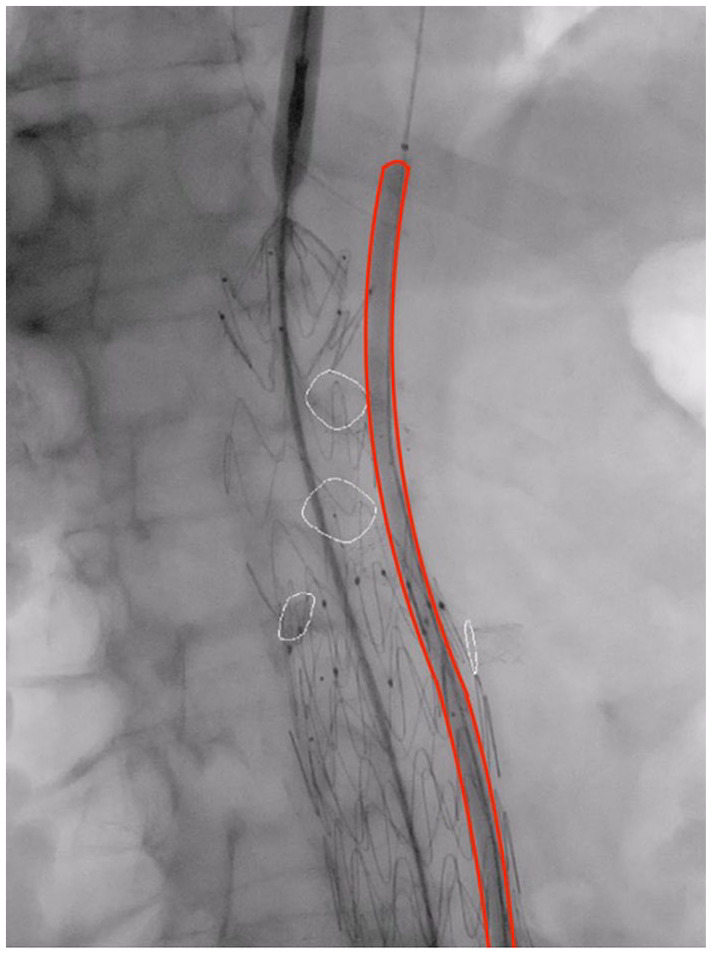
Schematic figure of gutter leak creation to diminish ischemia time during ISLF with 5 mm × 200 mm PTA balloon inflated along the stentgraft that is implanted across the visceral arteries.

**Figure 3.**
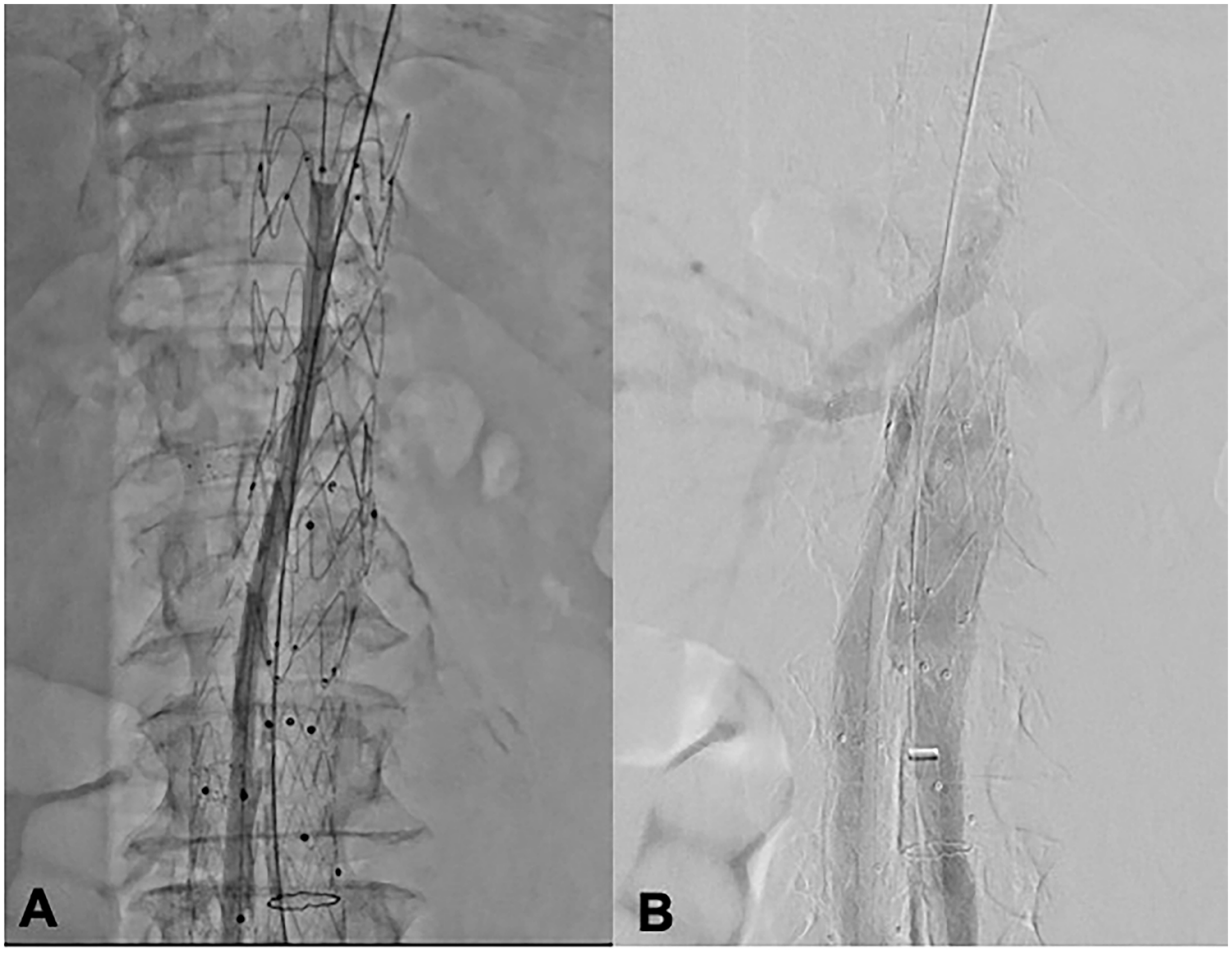
(A) Inflation of a 5 mm × 200 mm balloon parallel to the stentgraft that is implanted across the visceral arteries (Cook Alpha thoracic graft), in order to create gutter leak for perfusion of the visceral during in situ laser fenestration. (B) Angiogram with injection of iodine contrast in the aorta, showing perfusion of the SMA and renal arteries (right renal and SMA opacified, left renal vaguely opacified) through gutter leak along the balloon after implantation of the aortic stentgraft.

The procedure setup was similar to that used for patient #1. Furthermore, repeated automated CO_2_ injections (Angiodroid Srl, San Lazzaro, Bologna, Italy) were performed alongside the use of a limited volume of iodinated contrast agent due to a known severe chronic kidney disease. All visceral vessels were prestented. A 5 mm × 200 mm Armada angioplasty balloon (Abbott, Green Oaks, IL, USA) was advanced in the infrarenal aorta over a Rosen guidewire. Using fusion imaging, a 40 mm tubular Cook Zenith Alpha graft was advanced in position. Prior to deployment, the 5 mm × 200 mm angioplasty balloon was placed between the unreleased endograft and the aortic wall and inflated, with the intention to provide continuous perfusion to SMA and renal arteries while creating ISLF. The length of the balloon was chosen in order to allow for an antegrade and retrograde gutter endoleak from both ends. After stentgraft deployment, an angiogram with CO_2_ confirmed complete retrograde perfusion of the LRA and the SMA (Figures 2 and 3; Supplemental Video S2). Then the ISLF for all vessels was performed, starting with the RRA, followed by the SMA. The angioplasty balloon for the gutter endoleak was removed after SMA and RRA were stented and completely reperfused, after which the LRA ISLF, followed by the CT revascularisations, were performed. Completion angiography showed patent visceral bridging stents and no types I or III endoleak. CBCT imaging for completion control confirmed good technical results. The postoperative course was uneventful, with stable creatinine and lactate and prompt clinical recovery (Figure 1B).

## Discussion

In this technical note, we address one of the challenges encountered during the urgent management of pAA with the ISLF technique, namely reno visceral ischemia time. We propose two feasible techniques that can potentially reduce the risk of renovisceral malperfusion complications during ISLF procedures.

ISLF is an innovative technique that provides a tailored endovascular solution for various complex aortic pathologies.^6^ In the case of ISLF involving the visceral aorta, high technical success rates^2,5–7^ comparable to postoperative mortality and reintervention rate to elective CMD F-EVAR^2^ with low rates of mesenteric ischemia have been reported. The main drawback of this technique is still that the deployment of the aortic endograft leads to a temporary ischemia due to the coverage of all visceral ostia.^4^ Leger et al.^7^ in their experience on 20 patients with 50 successfully revascularized target vessels, reported 34 minutes of median ischemic time for SMA, 69 minutes for LRA, 73 minutes for RRA and 93 minutes for CT, with a 5% rate of mesenteric ischemia. Visceral ischemia times were consistent with those reported by Le Houérou et al.^2^ on 42 patients with 108 target vessels, describing median ischemic time of 7.5 minutes for the SMA, 48 and 50 minutes for LRA and RRA, respectively, and 125 minutes for the CT. As the ISLF technique constantly improves, the visceral ischemia time decrease consequently such as reported by our group^6^ with 7 minutes for the SMA (range: 4–12 minutes), 22 minutes for renals and 43.5 minutes for CT.

Despite the high success rate, both renal failure and mesenteric ischemia complications have been reported after ISLF. Leger et al.^7^ in their 20 patients experience reported a death on postoperative day 5 due to a presumed mesenteric ischemia, and one case of a patient needing in-hospital temporary dialysis.^7^ Le Houérou et al.^2^ in their experience on 42 patients reported a 13.6% acute renal failure rate (6 patients), requiring temporary dialysis in three cases; no mesenteric ischemia was reported.^2^ Our group^6^ reported two cases of transient acute kidney injury not requiring dialysis in a ruptured and in a symptomatic thoraco-abdominal aortic aneurysm (TAAA).^6^ Furthermore, a case of mesenteric ischemia requiring SMA thrombectomy and CT stenting with partial bowel resection was reported in a TAAA treated in a multistaged fashion.^8^

In our case of gutter endoleak creation with a 5-mm angioplasty balloon, SMA stenting was performed in 8 minutes, RRA stenting in 20 minutes, LRA stenting in 35, and CT stenting in 50 minutes, underlining that in this frame time, visceral organs were continuously perfused because of the endoleak creation via the space between the balloon and endograft. Of note, the balloon, which was positioned along the left border of the aorta, was removed before the LRA ISLF procedure. This was done to avoid the risk of distortion of the stentgraft after ISLF creation in this area, which would have been a risk if the balloon had been removed after the ISLF procedure. If the balloon placement along the aortic wall is in the wrong position (eg, dorsal, not allowing adequate perfusion the viscera), some rescue maneuvers could be considered, such as the 8Fr sheath placement in the target viscera, which is an easily available alternative. The balloon location depends on the iliac and aneurysm anatomy, but it is likely along the contralateral wall of the aorta compared to the iliac where it is inserted. In the current case, CO_2_ angiogram was used for the assessment of visceral perfusion through the gutter endoleak. However, it should be noted that CO_2_ has better diffusion capacity than iodinated contrast. Hence, it may possibly overestimate the amount of perfusion.

Several solutions have been previously proposed to reduce ischemia time for the renal arteries during ISLF. Le Houérou et al.^5^ reported using a stent graft undersized at the level of the visceral aorta level to prevent temporary ischemia. Tse et al.^9^ describe the use of a catheter with the double aim of marking the target vessel for the ISLF and cold-perfusing the renal arteries. Bismuth et al.^10^ performed ISFL with a constrained stent graft to allow perfusion to the renal arteries, as Wang et al.^11^ reported a case of ISLF for the RRA performed with the bare stent region unreleased, ensuring continuous perfusion throughout the procedure. All these techniques seem to be feasible in the case of juxtarenal aneurysms.

In the case of pAA, further efforts are required in order to reduce the renovisceral ischemia time. Our group^3,12^ developed an off-the-shelf CMD stent graft with a single 8-mm fenestration (Cook) that is primarily designed to enable continuous perfusion of the SMA during ISLF. This eliminates the issue of SMA-related bowel ischemia and significantly reduces the time to revascularization of the CT and the renal arteries.^3,12^ However, this CMD solution is not suitable for treatment of type IA endoleak after EVAR.

The techniques described in this note have several advantages: (1) they are easy to perform, even in urgent/emergent settings; (2) they don’t require ad hoc devices; (3) they do not affect the rest of the ISLF procedure. Since the above presented two cases, we have used the gutter endoleak technique in several ISLF cases with similar results. However, as for the ISLF procedure itself, the use of these techniques should be limited to well-experienced centers dealing with the treatment of complex aortic pathologies. While the described use of gutter endoleak for renovisceral perfusion is simple and applicable in most cases, it may be less applicable in cases of free rupture or presence of shaggy aorta with risk for embolization.

## Conclusions

The techniques reported in this note can be considered safe and feasible ways to maintain visceral perfusion during ISLF for pAA. However, the short—and long-term effects of these techniques need to be studied in larger patient cohorts.
